# Probiotics for Prevention of Severe Necrotizing Enterocolitis: Experience of New Zealand Neonatal Intensive Care Units

**DOI:** 10.3389/fped.2020.00119

**Published:** 2020-04-07

**Authors:** Michael P. Meyer, Sharon S. W. Chow, Jane Alsweiler, David Bourchier, Roland Broadbent, David Knight, Adrienne M. Lynn, Harshad Patel

**Affiliations:** ^1^Neonatal Unit, KidzFirst, Middlemore Hospital, Auckland, New Zealand; ^2^Department of Paediatrics: Child and Youth Health, University of Auckland, Auckland, New Zealand; ^3^Faculty of Medicine, University of New South Wales, Sydney, NSW, Australia; ^4^Neonatal Unit, Auckland City Hospital, Auckland, New Zealand; ^5^Neonatal Unit, Waikato Hospital, Hamilton, New Zealand; ^6^Paediatrics, Dunedin Hospital, Dunedin, New Zealand; ^7^Neonatal Unit, Christchurch Women's Hospital, Christchurch, New Zealand; ^8^Neonatal Unit, Wellington Hospital, Wellington, New Zealand

**Keywords:** probiotics, necrotizing enterocolitis, preterm, prevention, late onset sepsis

## Abstract

**Introduction:** Necrotizing enterocolitis (NEC) affects mainly preterm infants, has a multifactorial etiology and is associated with intestinal dysbiosis and disordered immunity. Use of probiotics for prophylaxis is beneficial with studies indicating reduction in NEC ≥ stage 2, late onset sepsis (LOS) and mortality. However, not all studies have shown a reduction, there are questions regarding which probiotic to use, whether infants <1,000 g benefit and the risk of probiotic sepsis. All neonatal intensive care units in New Zealand (NZ) use probiotics and contribute to an international database (Australian and New Zealand Neonatal Network or ANZNN).

**Objective:** To use ANZNN data to investigate the experience of NZ neonatal units with probiotics for NEC prevention in a setting where the baseline incidence of severe NEC was low, to compare results of 2 commonly used probiotic regimes and report on the extremely low birth weight subgroup.

**Method:** Outcomes before (Pre group 2007–2010) and after (Probiotic group 2013–2015) starting routine probiotics for preterm infants <1,500 g or <32 weeks were compared. Clinicians reviewed cases to ensure they met database criteria. Five units used Infloran (*Bifidobacterium bifidum* and *Lactobacillus acidophilus)* and 1 unit used *Lactobacillus GG* (LGG) and bovine lactoferrin (bLF).

**Results:** Four thousand five hundred and twenty nine infants were included and Pre and Probiotic groups were well-balanced with regard to gestation, birth weight and gender. The incidence of NEC in the Probiotic group was 1.6 and 2.7% in the pre group (corrected OR 0.62 CI 0.41–0.94). There was one case of probiotic sepsis. There was no significant difference between the Infloran and LGG/bLF combinations in regard to observed NEC rates. Late onset sepsis rates were significantly lower in the Probiotic group (*p* < 0.01).

**Conclusions:** Introduction of probiotics for preterm infants in NZ has been associated with significant reductions in NEC and late onset sepsis.

## Introduction

Necrotizing enterocolitis (NEC) is a condition with intestinal dysfunction associated with bowel wall necrosis, inflammation and, at times, perforation (1) It predominantly affects preterm infants but no neonatal group is exempt. Clinical presentation is varied with a staging system (Bell's modified criteria) used to assist classification. Severe cases (modified Bell's stage 2 or more) have combinations of abdominal distension, pneumatosis, portal venous gas and perforation (2–4). Morbidity including long term sequelae and mortality (30–40%) are high (5). An incidence of over 10% has been reported in infants <1,500 g or <32 weeks (6) but is considerably lower in New Zealand (NZ) and Australian units with a rate of 4.4% noted in a recent large study (7) and a rate of 3% reported from one NZ unit (8).

Considerable evidence exists of a beneficial effect from probiotic supplementation in preterm infants for prevention of stage 2 or more NEC. Recent meta-analyses of randomized clinical trials have reported a 43% reduction with a risk ratio of 0.57 (0.47–0.70) from 29 trials with over 4,000 preterm infants receiving probiotic supplements. Late onset sepsis (LOS) and mortality were also significantly reduced (9). Likewise, meta-analysis of observational studies showed similar improvements in NEC, LOS and mortality (9, 10). Significant negative effects were not reported (9). In spite of these seemingly powerful results, many remain unconvinced of the effectiveness of probiotics. Heterogeneity exists in the study populations, the probiotics used and their administration. Randomized trials have not shown a reduction in NEC in infants <1,000 g (but confidence intervals do not rule this out), and a recent large trial in the United Kingdom showed a non-significant result, but did not rule out benefit (11, 12). Head to head comparisons between probiotics have not been carried out.

Studies from neonatal networks can add to the body of knowledge on probiotics, may include large numbers of infants and have more homogeneity in regard to probiotics used. There have been a number of such studies, and, in general, the results have been consistent with the meta-analyses of randomized trials (13, 14). All NZ and Australian level 3 neonatal units report standardized data to a central network (Australian and New Zealand Neonatal Network or ANZNN). Across NZ neonatal intensive care units (NICUs), probiotics have been in use from 2011. Most units in NZ use Infloran (*Lactobacillus acidophilus* and *Bifidobacterium bifidum)*, while one unit uses the probiotic *Lactobacillus GG* (LGG) and bovine lactoferrin (bLF). Our aim was to compare cases of severe NEC (stage 2 or more) across NZ in a setting of very low baseline rates before and after use of probiotics, to compare results with different probiotic preparations, to perform subgroup analysis in infants <1,000 g and report other neonatal outcomes, particularly late onset sepsis.

## Materials and Methods

A questionnaire was sent to NZ level 3 NICUs in 2016 to determine whether probiotics were in routine use, the indication(s) for use, population targeted, strain(s) and dose of probiotic, starting and stopping criteria, any reasons for withholding doses, any side effects noted, cases of probiotic sepsis, and any periods of involvement in a randomized trial of probiotics (15).

All NZ level 3 NICUs responded to the questionnaire and from the results the intervention (Probiotic) cohort (2013–15) was chosen. Prior to 2013, not all NICUs used probiotics and some were involved in randomized trials of probiotic use. The cut off for inclusion (2015) was chosen because of erratic availability of one of the probiotics (Infloran) after this date, which resulted in different probiotic preparations being used. The pre-intervention (pre-probiotic) cohort was chosen to include years prior to the probiotic studies (2007–2010). The population of infants receiving probiotics across the 6 centers was <32 weeks and/or <1,500 g.

One unit used *Lactobacillus GG* (LGG), one capsule, at a daily dose of 6 × 10^9^colony-forming units (Dicoflor60 (ATCC 53103) Dicofarm SpA, Rome, Italy) and bovine lactoferrin (bLF), one sachet, 100 mg daily (LF100; Dicofarm SpA). The other 5 units used Infloran (SIT Laboratorio Farmaceutico, Mede, Italy) 250 mg capsules containing 10^9^colony forming units (CFU) *Lactobacillus acidophilus* (ATCC 4356) and 10^9^ CFU *Bifidobacterium bifidum* (ATCC 15696). All units using Infloran gave one capsule daily for infants <1,500 g; in one unit this was given as a half capsule twice daily while in another unit infants >1,500 g received 2 capsules daily. Dosing was continued for 4–6 weeks or until 34 weeks corrected gestation in 4 of the 6 units; in the other 2 it continued until 36 weeks or discharge. In all cases probiotics were started when trophic feeds were commenced with the added proviso in 3 units that the feeds had been tolerated for 24 h (only one of the hospitals had a donor milk bank), For study purposes, it was assumed that all eligible infants received the probiotic according to local guidelines; individual medication charts were not reviewed.

Approval for the audit was obtained from the NZ ethics committee (HDEC Northern region), from the individual NICUs and the study met ANZNN guidelines for data use (available on line at https://www.anznn.net/research/researchrequest). De identified data was obtained for infants <32 weeks and/or <1,500 g. The network has defined criteria for the diagnosis of Bell's stage 2 or more NEC (2, 4, 16). These are consistent clinical signs plus at least one of the following: pneumatosis, portal vein gas, diagnosis at surgery or post mortem, fixed dilated loop on serial x-rays, abdominal cellulitis and palpable abdominal mass. Histologic diagnosis required the presence of necrosis; where there was perforation without evidence of necrosis this was regarded as spontaneous intestinal perforation (SIP) and not classified as NEC and not included in this analysis. Case histories were reviewed by local neonatologists to ensure ANZNN criteria for stage 2 or more NEC were met. We collected data on LOS in the same population. This was according to the network definition (16) which was blood or cerebrospinal fluid culture positive infection with bacteria or fungi not deemed to be contaminants. Maternal demographic data available from the network included maternal age, parity, ethnicity, presenting antenatal problem (preterm labor, hypertension in pregnancy, antepartum hemorrhage, suspected intra uterine growth restriction, fetal compromise, fetal malformation), inborn (yes/no), delivery method (vaginal or cesarean section), intrapartum antibiotic use (yes/no), antenatal steroid use (complete, incomplete, none), plurality (multiple pregnancy yes/no). Infant data included birth weight, gestation (based on first day of last menstrual period or early ultrasound at 8–10 weeks or best estimate if other measures not available), sex, Apgar score at 1 and 5 min, worst cord base deficit, admission temperature, major congenital malformation, duration of CPAP, high flow, or positive pressure ventilation, chronic lung disease (defined as respiratory support at 36 weeks corrected age), home oxygen use, days to full oral feeds, days to regain birth weight, breast milk at onset of feeding (yes/no), necrotizing enterocolitis (yes/no), SIP (yes/no) LOS (yes/no), retinopathy of prematurity (ROP) stage, ROP treated surgically (yes/no), intraventricular hemorrhage (IVH) grade, death during hospital admission (yes/no). https://www.anznn.net/dataresources/datadictionaries.

Categorical data was analyzed using chi square, Fishers exact test and odds ratios with confidence intervals, continuous data with the *t*-test if parametric or Mann Whitney U if non-parametric. Univariate statistics were used in the first instance to determine associations between covariates and outcomes. Following this, multivariate logistic regression was carried out and confounders were removed one-by-one in a backward selection process if the *p*-value was <0.1. Model summary characteristics and correlation matrices were used for model selection and the best parsimonious model developed for the outcome of interest e.g., NEC, LOS, IVH (IBM SPSS Statistics for Windows, Version 23.0. Armonk, NY). Where missing data accounted for 1–2% of total cases, the existing raw data was used. For other data regarded as missing at random, multiple imputation with 5 imputed data sets was used.

## Results

**Figure 1:**
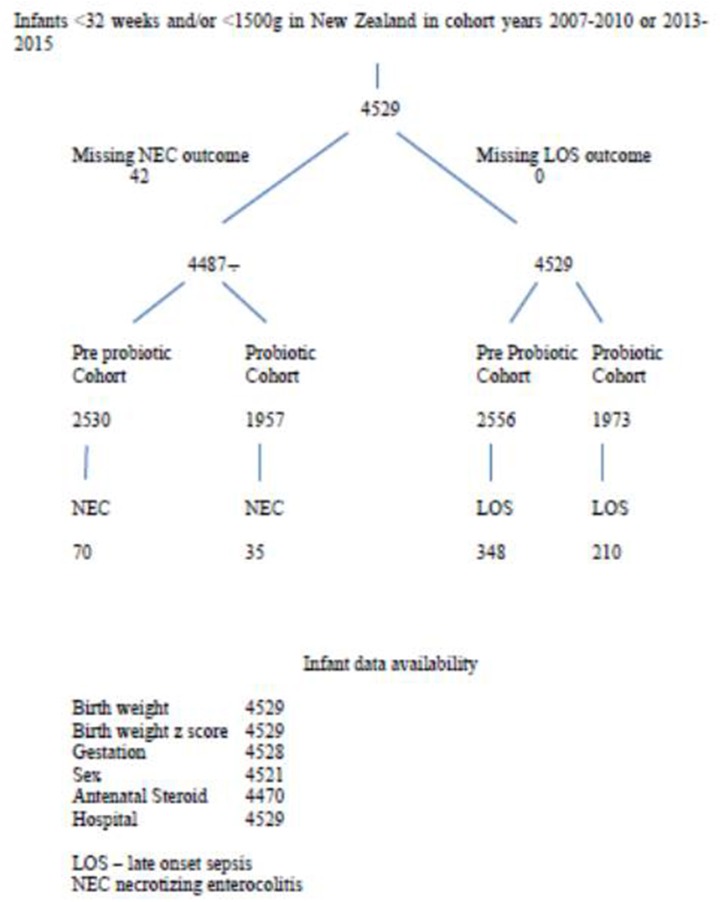
Flow diagram for necrotizing enterocolitis (NEC) and late onset sepsis (LOS).

There were 4,529 infants in the database and 3,899 mothers. Patient flow for the study is shown in Figure 1. Comparing variables between pre-probiotic and probiotic cohorts, there were significant differences in some antenatal demographics with lower percentages of infants exposed to antenatal complications in the earlier cohort including less intra partum antibiotic use (Table 1). There were also differences in ethnicity and percentage of singletons.

**Table 1:** Antenatal variables for preterm infants in the pre-probiotic and probiotic cohorts.

	**Pre-probiotic cohort**	**Probiotic cohort**	**OR (95% CI)**
Years	2007–2010	2013–2015	
Number	2,556	1,973	
Mothers	2,173	1,726	
PPROM %	19.2	310	0.53 (0.45–0.61)
PTL %	56.8	62.3	0.79 (0.70–0.89)
PET %	13.3	20.6	0.59 (0.50–0.69)
APH %	18.2	26.3	0.62 (0.54–0.72)
Antenatal IUGR %	12.5	18.8	0.62 (0.52–0.73)
Fetal distress %	18.4	34.3	0.43 (0.38–0.50)
Intrapartum antibiotics %	45.7	49.8	0.84 (0.74–0.95)
No antenatal steroids %	12.1	10.0	1.23 (1.02–1.49)
Incomplete antenatal steroids %	26.5	25.3	1.11 (0.96–1.26)
Complete antenatal steroids %	61.4	64.7	0.87 (0.77–0.98)
Singleton %	71.4	75.1	0.83 (0.72–0.95)
Ethnicity Asian number (%)	256 (10.3)	305 (15.6)	0.62 (0.51–0.74)
Caucasian n umber (%)	1363 (54.9)	857 (43.9)	1.55 (1.37–1.75)
Pacific Island number (%)	226 (9.1)	245 (12.5)	0.69 (0.57–0.84)
Maori number (%)	569 (22.9)	448 (22.9)	1.00 (0.86–1.15)
Inborn %	91.7	92.6	0.87 (0.70–1.09)
Vaginal birth %	61.3	60.2	0.95 (0.84–1.07)

*PPROM preterm pre labor rupture of membranes*.

*PTL, preterm labor; PET, pre eclampsia; APH, Antepartum hemorrhage; IUGR, intrauterine growth restriction; OR, odds ratio*.

Infant demographics (birth weight, gestation, sex, and birth weight z score) were not significantly different between cohorts although there were differences in the maximum recorded base excess, Apgar scores and admission temperatures (Table 2). Breast milk was more likely to be used at onset of feedings in the probiotic cohort.

**Table 2:** Univariate statistical comparison of pre-probiotic and probiotic cohorts.

	**Pre-probiotic cohort**	**Probiotic cohort**	***p*-value**	**Odds ratio 95% CI**
Number	2,556	1,973		
Birth weight mean (SD)	1,227 (373)	1,235 (381)	0.32	
Gestation median (IQR)	29 (4)	29 (4)	0.84	
Male sex%	54	53.6	0.74	
Worst base excess (SD)	−3.9 (4.7)	−4.3 (4.5)		0.37 (0.09 to 0.66)
Apgar 1 min (IQR)	6 (5–8)	6 (4–8)	0.003	
Apgar 5 min (IQR)	9 (8–9)	8 (7–9)	<0.001	
Admission temperature°C mean (SD)	36.38 (0.73)	36.48 (0.79)	<0.001	
Breast milk at onset %	81.9	93.4		0.32 (0.26 to 0.40)
Birth weight z score mean (SD)	−0.095 (1.105)	−0.064 (1.118)	0.36	
NEC number (%)	70 (2.7)	35 (1.8)		1.58 (1.04 to 2–6)^*^
LOS number (%)	348 (13.6)	210 (10.6)		1.32 (1.10 to 1.59)^*^

*CI, confidence interval*;

* *, unadjusted; NEC, necrotizing enterocolitis; LOS, late onset sepsis*.

There were differences between infants with NEC and those without when compared with univariate statistics (Table 3). Various antenatal complications were associated with NEC as was the use of intra partum antibiotics. However, antenatal steroid use, ethnicity and hospital site were not significantly associated with NEC. Birth weight and gestation were significantly lower in the NEC cases.

**Table 3:** Univariate associations with necrotizing enterocolitis (NEC) Stage 2 or more.

	**NEC**	**No NEC**	**OR**	***p*-value**
			with 95% CI	
Maternal age median (IQR)	29 (24–33)	30 (24–34)		0.34
PPROM %	25.3	23.5	1.1 (0.68–1.77)	
Preterm labor %	71.4	58.8	1.74 (1.12–2.72)	
Pre-eclampsia %	17.2	16.2	1.07 (0.62–1.84)	
Antepartum hemorrhage %	32.2	21.4	1.76 (1.13–2.74)	
Antenatal IUGR %	6.8	15.5	0.41 (0.18–0.87)	
Fetal distress %	15.5	25.5	0.54 (0.30–0.94)	
Intra partum antibiotics %	60.8	47.4	1.72 (1.12–2.63)	
Antenatal steroid none/any %	9.0	11.2	0.78 (0.39–1.56)	
Antenatal steroid complete %	70.3	70.3	0.99 (0.63–1.58)	
Ethnicity	–	–		0.33
Hospital	–	–		0.11
Out born %	6.8	7.8	1.16 (0.53–2.51)	
Vaginal birth %	44.6	38.9	1.26 (0.85–1.88)	
Multiple birth %	16.5	27.2	0.53 (0.31–0.89)	
Birth weight mean (SD)	909 (±265)	1,238 (±376)		<0.001
Gestation weeks (IQR)	26 (25–28)	29 (27–31)		<0.001
Male sex %	62	54	1.41 (0.92–2.14)	
Worst base excess	−5.65 (4.0)	−4.03 (4.6)	<0.001	
Apgar 1 min median (IQR)	6 (4–7)	6 (5–8)		0.026
Apgar 5 min median (IQR)	8 (7–9)	9 (7–9)		0.014
Admission temperature°C mean (SD)	36.22 (0.87)	36.41 (0.76)		0.119
Birth weight z score mean (SD)	−082 (1.11)	−081 (1.11)		0.993
Breast milk at onset %	83	87	0.73 (0.43–1.23)	

*PPROM, preterm pre labor rupture of membranes; IUGR, intrauterine growth restriction*.

*SD, standard deviation; IQR, interquartile range; OR, odds ratio*.

The multivariate regression model for NEC was derived initially from the significant univariate factors (see above). Many of the antenatal associations noted in Table 3 appeared to be related to birth weight or gestation and were no longer significant in the multivariate model. Exposure to intra partum antibiotics was associated with NEC in the univariate model but there was missing data in 11.5% so this was not included in the multivariate analysis. Multiple imputation with 5 datasets was carried out for missing worst base excess data. Data on breast feeding at discharge was only available in 50% of cases, so this was not further analyzed.

**Figure 2:**
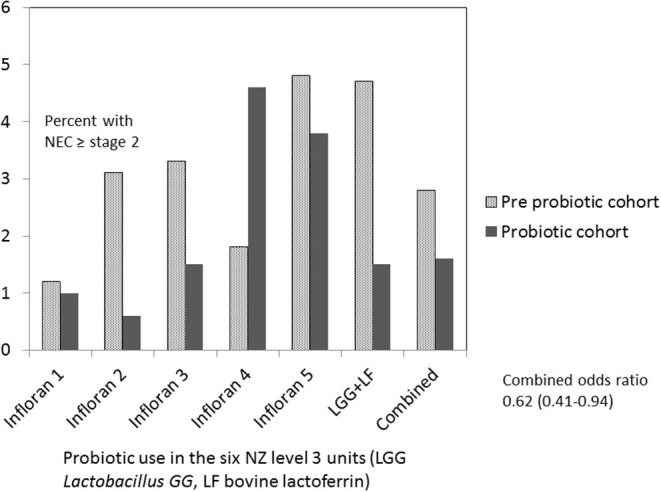
Necrotizing enterocolitis (NEC) <1,500 g or <32 weeks in New Zealand level 3 units before and after probiotics commenced.

Five of six hospitals noted a decline in NEC stage 2 or more associated with probiotic use, however, in one hospital the rate increased (Figure 2). Numbers of patients included at each site in the pre probiotic and probiotic cohorts were as follows: Infloran 1 537/396, Infloran 2 402/207, Infloran 3 764/501, Infloran 4 485/293, Infloran 5 171/110, LGG+LF 197/466. The overall rate in New Zealand declined from 2.7 to 1.8% in the <32 weeks and/or <1,500 g cohort. Using multivariate logistic regression the adjusted odds ratio was 0.62 (95% CI 0.41–0.94) in the probiotic cohort. Hospital site was not a significant contributor to the final model, whilst birth weight, birth weight z score, the cohort to which the baby belonged and male sex were all significant independent factors (Table 4). Gestation was less predictive than birth weight and adding it to the model did not improve it. For the final model, the chi square omnibus test for model coefficients was 114 (*p* < 0.001), and Hosmer and Lemeshow test chi-square 5.66 (*p* = 0.69). Additional covariates did not have a significant Exp (B) or exponentiated coefficient.

**Table 4:** Multivariate associations with necrotizing enterocolitis and late onset sepsis.

**NEC**		**Corrected odds ratios**	**95% CI**	***p-*value**
	Birth weight/100 g	0.71	0.66–0.76	<0.001
	Birth weight z score	0.66	0.54–0.81	<0.001
	Male	1.68	1.12–2.52	0.012
	Probiotic cohort	0.62	0.41–0.94	0.024
NEC	Infloran/LGG bLF	1.23	0.7–2.17	0.47
LOS				
	Gestation/week	0.64	0.61–0.67	<0.001
	Probiotic cohort	0.72	0.59–0.88	0.001
	PPROM	1.54	1.20–1.98	0.001

*NEC, necrotizing enterocolitis; LOS, late onset sepsis bLF bovine lactoferrin*.

*PPROM, preterm pre labor rupture of membranes*.

**Figure 3:**
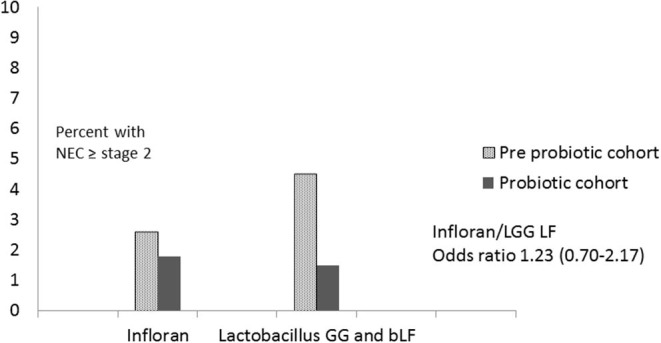
Comparison of Infloran with Lactobacillus GG (LGG) and bovine lactoferrin (bLF) for necrotizing enterocolitis (NEC) in New Zealand level 3 units.

Comparing results for the different probiotic regimens, there was a reduction in NEC in both groups and the corrected odds ratios showed a non-significant difference between them (OR 1.23 CI 0.70–2.17; *p* = 0.47 for Infloran/LGG bLF), with relatively wide confidence levels (Figure 3).

There was no significant difference in NEC rates between the cohorts for infants <1,000 g, and confidence intervals again were relatively wide (Table 5).

**Table 5:** Other neonatal outcomes in pre-probiotic and probiotic cohorts.

	**Pre-probiotic cohort**	**Probiotic cohort**	**OR (95%CI)**	***p-value***
Intubated at delivery %	37.9	36.6		0.37
IPPV hrs mean (SD)	184 (296)	161 (282)		0.001
CPAP hrs mean (SD)	552 (637)	552 (587)		0.27
High flow hrs mean (SD)	–	469 (380)		
Days to regain birth weight (SD)	10.25 (5.25)	9.15 (4.2)		<0.001
Chronic lung disease %	14.6	27.6	0.45 (0.39–0.52)	
Home oxygen %	6.20	9.73	0.61 (0.49–0.77)	
Treated ROP %	3.80	2.83		0.15
In-hospital mortality %	7.68	7.82		0.87
IVH any %	20.38	21.60	0.93 (0.78–1.10)	
IVH grade 3 or 4 %	4.60	7.04	0.70(0.49–1.00)^*^	
NEC <1,000 g %	5.9	4.8	1.25 (0.77–2.04)^#^	
Late onset sepsis <1,000 g %	29.2	24.3	1.34 (1.04–1.72)^$^	

*IPPV, intermittent positive pressure ventilation; hrs, hours; CPAP, continuous positive airway pressure; ROP, retinopathy of prematurity; IVH, intra ventricular hemorrhage; NEC, necrotizing enterocolitis; SD, standard deviation; OR, odds ratio; CI, confidence interval*.

* *Corrected for gestation, antenatal steroid use, sex, birth weight z score, ethnicity, birth hospital*.

# *Corrected for birth weight, sex, birth weight z score*.

$ *Corrected for gestation, PPROM*.

Late onset sepsis rates declined significantly in the cohort receiving probiotics with a corrected odds ratio of 0.72 (0.59–0.88). Regression indicated significant factors associated with LOS were gestation, cohort and PPROM.

### Other Neonatal Outcomes

Other outcomes are shown in Table 5. There were differences between the cohorts in the management of respiratory disease with less use of mechanical ventilation and greater use of non-invasive support. There was a borderline increase in grade 3 and 4 IVH rates in the probiotic cohort. The increase in severe IVH was mostly in <25 week gestation infants (from 16.4 to 25.7% *p* = 0.045) and there was an increase in the percentage of infants <25 weeks in this cohort (from 6.8 to 8.1%; *p* = 0.04). 48% of those with severe IVH died.

## Discussion

This study reports results for NEC stage 2 or more across all level 3 NICUs in NZ, capturing de-identified network data over two different time periods before and after probiotic use. All NICUs report to a centralized database (ANZNN) with consistent definitions for NEC and patient demographics. It is apparent that NEC is a relatively rare disease in NZ (2.7% before commencement of probiotics) with a corresponding low baseline prevalence in the Australian units (16). Nevertheless, NEC remains an important disease with consistently high mortality and morbidity, need for surgery, prolonged length of stay and associated costs. The finding of a reduction in NEC stage 2 or more by 38% with an adjusted OR 0.62 (0.41–0.94) in the probiotic cohort in NZ is, therefore, important and indicates that even from a low baseline, further decreases can be expected in association with probiotic use. As an observational study, the strength of these findings is limited by potential confounding, but the results are consistent with those of meta analyses of randomized trials, other observational cohorts and network studies.

A recent meta-analysis of 29 randomized trials which compared probiotic use with placebo in very low birth weight infants, indicated a decrease in NEC with probiotic use with a risk ratio of 0.57 (0.47–0.70) without significant heterogeneity (9). Meta analysis of observational studies by the same authors showed similar findings, as did an earlier meta-analysis (10), with significant heterogeneity in both studies. Other network studies from Germany (10,890 preterm infants) and Canada (1,631 preterm infants) showed similar significant reductions in severe NEC (13, 14). In contrast to these results, however, in a national cohort of Finnish babies <32 weeks, there was no reduction in NEC in the center using *Lactobacillus GG* as probiotic (17). In our current study 5 of 6 NICUs reported a decline in NEC, but there was an increase at one. There were no obvious differences in terms of type of probiotic, its dose, age of commencement or duration of use. The probiotic cohort at this hospital was relatively small (about 300 infants) and the incidence of NEC (4.4%) was not high in relation to experience of many units in ANZNN (16). Although this difference is unexplained, other factors to consider include probiotic viability, storage of expressed breast milk and differences in case mix.

One of our goals was to compare NEC rates associated with use of Infloran and the combination of probiotic (LGG) and bLF. For mainly historical reasons, one unit used a combination of LGG and bLF while the other 5 units used Infloran. As shown in the results, the prevalence of NEC was similar across units in the historic cohort and a reduction was seen with both probiotic regimens with no significant difference between them. Deciding which probiotic (s) to use has been addressed in a number of publications using sub group analyses of randomized trials (9, 18, 19). *Lactobacillus GG* and *Bifidobacterium lactis* when used alone, were associated with significant reductions with narrow confidence intervals. Mixtures of two or more types of probiotics were associated with similar reductions and confidence intervals. Whilst use of *L reuteri* or *Saccharomyces* or *B breve* did not show significant reductions, the confidence intervals included the possibility of reductions between 8 and 29% (9). A recent meta analysis (20) found no difference between single or multistrain probiotic use and NEC. An excellent case has been made that the NEC reduction may not be strain specific but the mechanism of action could be shared by the wider probiotic genus (12) Most of the current data would tend to support this and the case for probiotic use continues to be overwhelming, even in the absence of head to head comparisons.

The use of bLF alone was shown in earlier studies to significantly reduce the NEC rate as was the combination of bLF and LGG (21). Lactoferrin is bifidogenic under certain conditions, and bLF enhanced growth of *B infantis in vitro*, which could potentially enhance the probiotic spectrum in supplemented infants (22). A Cochrane review and meta analysis (23, 24) indicated a beneficial effect of bLF on NEC rates, although the evidence was regarded as moderate to low grade. Results from the large ELFIN multicentre study (25) showed no reduction in LOS or NEC rates (although the study was underpowered for the NEC outcome). There are a number of potential reasons for these inconsistencies, including differences in the source and preparation of the bLF (21, 26, 27) and the intake of human lactoferrin in mother's own milk (28). Supplementation with bLF may be useful in a subgroup of preterm infants not receiving adequate volumes of mother's milk and this requires further study.

As noted in our results, NEC rates were not significantly reduced in Extremely Low Birth Weight (ELBW) infants in the NZ units. A similar finding was reported in a recent meta-analysis of randomized trials (9, 29). However, only one trial specifically enrolled ELBW infants and in another they were a pre specified subgroup. The NEC rate was not reduced but the meta-analysis was probably under powered (29). However, the summary statistics do not exclude a reduction in NEC and the meta-analysis showed other benefits such as reduced time to full oral feeds (29). An observational study included nearly 5000 ELBW infants and found a significant reduction in NEC (13). The observational data is compelling and our own data does not exclude benefit in the ELBW subgroup.

In our survey of NZ units, we noted variability in the timing of starting probiotics although commencement was early with trophic feeds. Duration of probiotic use also showed variation but the minimum use was more than 2 weeks. Subgroup data examined in meta-analyses have shown significant reductions in NEC rates when probiotics were started either with the first feed or after 48 h (29). Similarly, continuing for at least 14 days was associated with a reduction in NEC, although most studies reported use of 28 days or more (19).

The economic costs of NEC are difficult to accurately assess and variable estimates have been cited in the USA from US $43000 to US $398000 for surgical cases (30–33). In NZ, we are not aware of cost estimates, however, with a daily cost of around NZ $2 for probiotics, a number needed to benefit of 110 (prevalence reduced from 2.7 to 1.8%) and average length of stay of 50 days, this equates to a cost of NZ $11000 per case of NEC prevented, assuming probiotics are given from birth to discharge.

Unwanted effects of probiotics were not reported from the NZ units, although there was one case of probable probiotic sepsis with *Lactobacillus GG* in a 23 weeks gestation infant which has been reported previously (8). Although there are sporadic reports in the literature describing probiotic sepsis and implicating different probiotic strains, it appears to be a rare event as the larger network, observational and meta analyses have not reported cases (34, 35). It should be born in mind that anaerobic cultures are infrequently performed, so the number of cases could be underestimated. Ongoing safety and quality control at the point of probiotic production is important (36). In NZ probiotics are regarded as dietary supplements and are not subject to the same rigorous controls as other medicines. Batch testing has been recommended (37) and is carried out in some of the hospitals in NZ. Quality control is performed by the manufacturers and the products, including lactoferrin (which is subject to holder pasteurization) have “generally regarded as safe” or GRAS status. However, greater product surveillance by regulatory bodies would be beneficial.

Late onset sepsis rates were significantly reduced (by 25%) in the cohort receiving probiotics, as has been noted in meta-analyses of randomized trials and observational studies (9, 10, 29). In our study confounding by other changes in practice is possible with central line bundles of care being introduced in most units during this time period. Nevertheless, the data from randomized trials is robust and seems to be shared by different probiotic strains. There is uncertainty about the role of bLF, although there is potential benefit in cases where mother's milk supply is insufficient.

There were changes noted in other neonatal outcomes during the time period under study. Days to regain birth weight was significantly decreased. This could be related to changes in nutrition practices with increased protein intake being targeted in many units. Although studies have reported decreased time to full oral feeds in infants receiving probiotics (19), the ANZNN database did not collect this data for the whole period and we were unable to assess this outcome. There was increased use of CPAP and an increase of chronic lung disease in the probiotic cohort. These changes (as well as increased use of home oxygen), have been noted across the ANZNN network, started before probiotics were introduced (38), and based on logistic regression (results not shown) were not related to case mix. The causes are likely multifactorial and beyond the scope of this article.

While meta analyses of randomized studies showed reduced in-hospital mortality (with a recent updated study reporting a decrease of 1.6%), we did not detect any significant change (9). The borderline increase in grade 3 and 4 IVH coincided with changes in management of infants of borderline viability in NZ, with intensive care more likely to be offered in the period when probiotics were introduced (39). This shift in clinical practice may have affected results in regard to severe IVH and mortality.

### Limitations

Infants at one hospital received bLF in addition to the LGG probiotic. Although bLF is not a probiotic, it has been used with LGG in 2 studies reported in the Cochrane review and these studies are included in meta analyses of the effects of probiotics (9, 18–20). Therefore, there is international experience with this combination and we believe there is precedent for including these patients in the study.

Although the cohorts were well-matched in relation to infant characteristics such as birth weight and gestation, there were notable differences in antenatal and perinatal demographics. Many of the maternal variables (e.g., preterm labor, antenatal IUGR, antepartum hemorrhage, use of antibiotics) and perinatal characteristics (worst base excess, Apgar scores) indicated greater risk for NEC in the probiotic cohort On the other hand, there was an increased proportion of completed antenatal steroids amongst the women and more early use of breast milk in the probiotic cohort which may have mitigated this increased risk. However, based on the results of multivariate logistic regression, there was still a clear association between probiotic use and a decreased rate of NEC and this was minimally affected by confounders. We did not perform a time series analysis as data collected by ANZNN from 2001 to 2010 indicated that in the pre probiotic cohort the percentage of infants diagnosed each year with NEC, although showing some variation, was stable or slightly increased over the 10 year period (40), so that the prevalence of NEC had not already started to decrease before the probiotic intervention.

The ANZNN database does not include information on formula use, duration of breast feeding, gastric acid inhibitors or transfusions so that these potential confounders could not be further analyzed. Data on breast feeding at discharge was not able to analyzed because of missing information.

The case definition of NEC, even when confined to severe cases, has been recognized as being problematic, particularly as the clinical and radiographic changes may be subject to over or under reporting. In a recent Swedish study there was initial over reporting of >30 and 10% of cases were missed (41). To improve consistency, neonatologists from each center in NZ reviewed their own databases to ensure the ANZNN diagnostic criteria were met and to report cases that may have been missed.

## Conclusions

We noted a 38% reduction in cases of stage 2 or more NEC in NZ NICUs associated with the introduction of probiotics. This reduction was seen despite a very low background rate of severe NEC. We were able to compare results obtained with Infloran (*Lactobacillus acidophilus* and *B bifidum*) and *Lactobacillus GG* in combination with bovine lactoferrin and noted similar reductions with both regimes. At the same time cases of late onset sepsis were significantly decreased but in-hospital mortality was unchanged. There was one case of probiotic sepsis but no other unwanted effects from probiotics were apparent and their use is likely to be highly cost effective. These results are in keeping with the substantial evidence of benefit obtained from previous studies, provide support for routine probiotic use and indicate comparable results with the different combinations used in NZ NICUs.

## Data Availability Statement

The datasets generated for this study will not be made publicly available. The data was collected as de-identified patient data and as such is subject to the ANZNN data repository rules which do not permit data sharing outside of the network. Requests to access the datasets should be directed to Michael P. Meyer, michael.meyer@middlemore.co.nz.

## Ethics Statement

The studies involving human participants were reviewed and approved by Health and Disability Ethics Committee Northern region, New Zealand. Written informed consent from the participants' legal guardian/next of kin was not required to participate in this study in accordance with the national legislation and the institutional requirements.

## Author Contributions

MM, SC, JA, DB, RB, DK, AL, and HP helped design the study. SC supplied and collated the de identified data from ANZNN. MM was responsible for data analysis and writing the first draft of the paper. JA, DB, RB, DK, AL, and HP checked the NEC data at each hospital. All authors had input into writing the paper and provided critical evaluation of results.

### Conflict of Interest

The authors declare that the research was conducted in the absence of any commercial or financial relationships that could be construed as a potential conflict of interest.
